# Liaw's Ellipse Anteversion Method for Distinguishing Acetabular Component Retroversion from Anteversion on Plain Radiographs

**DOI:** 10.1111/os.13902

**Published:** 2023-11-20

**Authors:** Chun‐Hao Lin, Wei‐Cheng Chen, Pei‐Wei Weng, Yu‐min Huang, Chen‐Kun Liaw

**Affiliations:** ^1^ Department of Orthopaedics, Shuang Ho Hospital Taipei Medical University New Taipei Taiwan; ^2^ Department of Orthopaedics, School of Medicine, College of Medicine Taipei Medical University Taipei Taiwan; ^3^ International PhD Program in Biomedical Engineering, College of Biomedical Engineering Taipei Medical University Taipei Taiwan; ^4^ Research Center of Biomedical Devices Taipei Medical University Taipei Taiwan; ^5^ Research Center of Biomedical Device College of Biomedical Engineering, Graduate Institute of Biomedical Optomechatronics, Taipei Medical University Taipei Taiwan; ^6^ TMU Biodesign Center Taipei Medical University Taipei Taiwan

**Keywords:** Acetabulum, Ellipse Method, Liaw's Version, Radiographs, Retroversion, Total Hip Arthroplasty

## Abstract

Improper acetabulum component position is a significant risk factor for postoperative dislocation after total hip arthroplasty. Several radiographic two‐dimensional methods exist for measuring acetabulum component anteversion, but they cannot distinguish between anteversion and retroversion. “Liaw's version,” initially proposed as a simple mathematical standardized two‐dimensional method, was modified to the computerized ellipse method, proving superior accuracy to traditional two‐dimensional methods. In this article, we demonstrated its application in detecting and measuring retroverted acetabulum component. We obtained anteroposterior pelvis radiographs from a patient undergoing total hip arthroplasty on the day of surgery and 2 weeks postoperatively. The computerized ellipse method was used to measure the acetabulum component orientation. Upon comparison, the difference between θ assigned to be retroverted (9.52–8.56 = 0.96) is much smaller than the difference between θ assigned to be anteverted (23.81–18.86 = 4.95), leading us to determine retroversion. This was further confirmed by computed tomography at the 6‐week follow‐up. We propose that using the computerized ellipse method to measure Liaw's version can be a valuable tool in identifying acetabulum component retroversion on anteroposterior radiographs during routine postoperative follow‐up and retrospective assessments of total hip arthroplasty patients.

## Graphical abstract

The computerized ellipse method, as far as we know, is the first two‐dimensional radiographic method capable of distinguishing retroverted acetabular components in plain radiographs. The Elliversion software facilitates a quick and easy measurement process. Although further cases are necessary to determine the reliability of this method, it can be suggested as a tool for detecting acetabular component retroversion on anteroposterior radiographs during routine postoperative follow‐up and retrospective reviews of patients who have undergone THA.

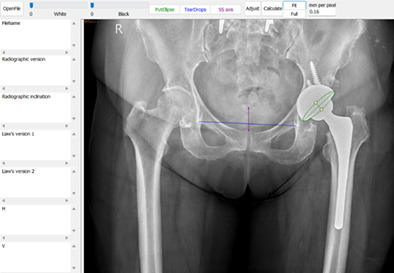

## Introduction

Total hip arthroplasty (THA) is a reliable and effective treatment option for end‐stage hip disease, providing satisfactory long‐term outcomes. Nevertheless, early complications such as dislocation or infection continue to be a concern.^1^ Postoperative dislocation is one of the most common reasons for revision surgery, and the incidence after primary THA ranges from 0.3% to 3%.^2^ Improper acetabulum component position is one of the major risk factors for postoperative dislocation.^3^, ^4^, ^5^, ^6^, ^7^ It also predisposes to component loosening, accelerated polyethylene wear, poor range of motion, and impingement.^8^, ^9^, ^10^, ^11^ Therefore, measuring the position of the acetabulum component is a crucial aspect of the postoperative follow‐up evaluation. The orientation of acetabular cup is defined as inclination and anteversion.^12^ Several two‐dimensional methods have been proposed to measure anteversion angle of the acetabular cup on plain radiographs.^3^, ^13^, ^14^, ^15^, ^16^, ^17^ However, one issue that remains unresolved is that none of the currently available two‐dimensional methods can distinguish retroversion from anteversion in plain radiographs.

“Liaw's version” was initially proposed as a simple mathematical standardized two‐dimensional method for measuring the acetabulum component anteversion following THA, using anteroposterior radiographs of the pelvis.^18^ The standardized radiographic anteversion angle was defined as the angle between pelvis radiographic axis (from the mid‐point of sacrococcygeal junction to the center of upper pole of symphysis pubis) and the plane of acetabulum component. In 2013, the authors developed the Elliversion software using the computerized ellipse method to measure Liaw's version solving the problem of difficultly in identifying the endpoints of the long and short axes of the ellipse.^19^ With the assistance of the Elliversion software, the speed and ease of measurement can be improved. Meanwhile, the authors also prove it to be a precise and concise method with good intra‐ and interobserver reliabilities.^20^ The most important aspect is that, unlike other two‐dimensional methods, the original standardized equation of Liaw's version took both anteversion and retroversion into consideration. After the calculation through the standardization process, two version angles would be generated, with θ assigned as positive (anteversion) and negative (retroversion), respectively. By comparing the measurements from two different anteroposterior radiographs of the pelvis, we can determine whether the acetabulum component is anteverted or retroverted. In this study, we demonstrated the application of the computerized ellipse method in the detection and measurement of retroverted acetabulum component.

## Methods

This study was approved by the TMU‐Joint Institutional Review Board (TMU‐JIRB) under registration number N202005040. The pelvis anteroposterior radiographs and CT images of a patient undergoing total hip arthroplasty were obtained from the picture archiving and communication system of our institute.

### Measurement with Elliversion Software

**FIGURE 1:**
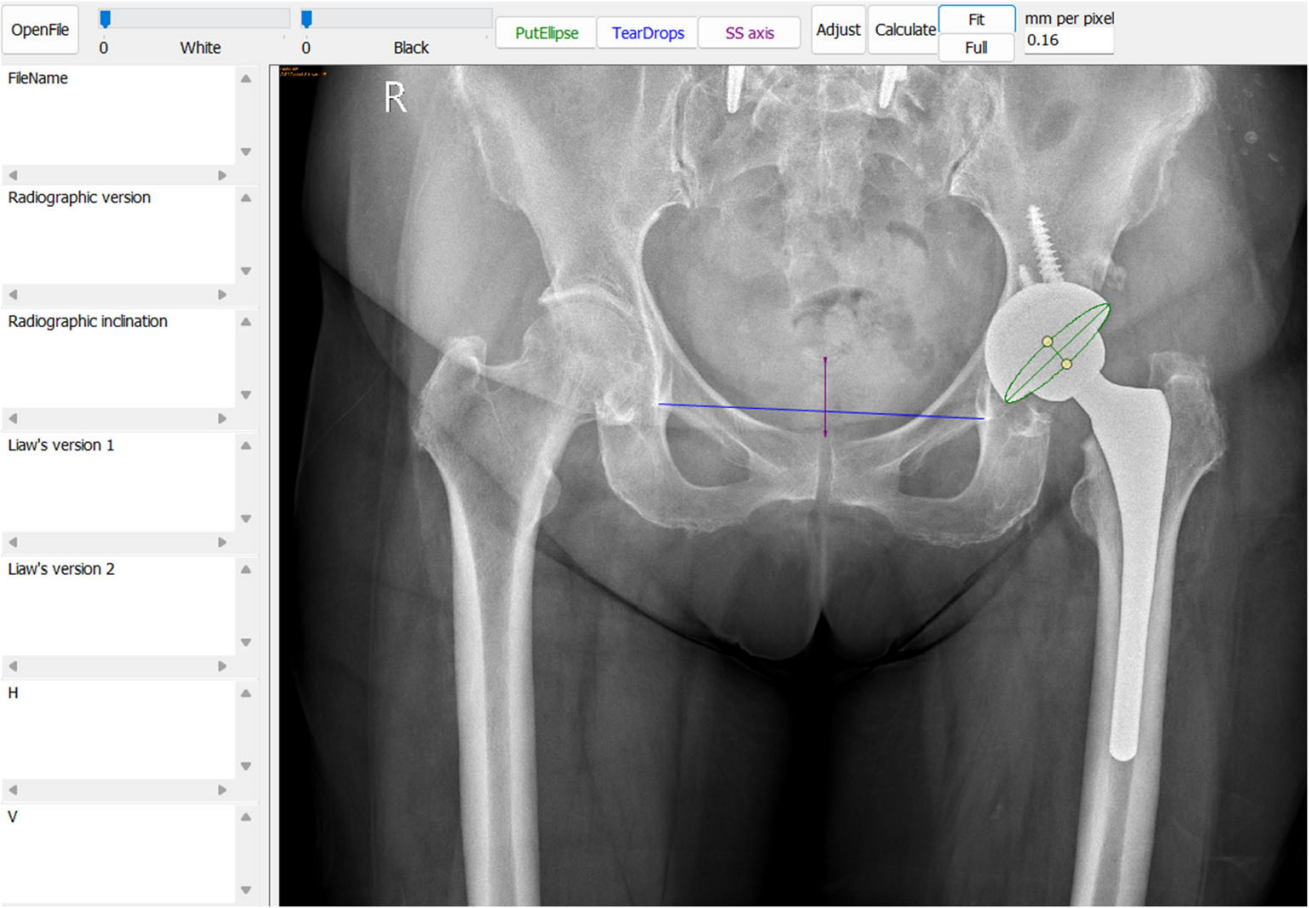
Using computerized ellipse method to measure Liaw's version by Elliversion software. The long and short axes of the ellipse along with the elliptical shell are shown in green. The blue line represents inter‐teardrop line as the horizontal reference of pelvis. The purple line (from center of sacrococcygeal junction to mid‐point of upper pole of pubic symphysis) represents the radiographic pelvis axis.

The anteroposterior radiograph of the pelvis without any requirements for the projection position was loaded into the Elliversion software (independently developed by the corresponding author on the Microsoft Windows platform). First, we manually generated an ellipse approximating the acetabulum cup opening. Users could adjust the long and short axes of the ellipse for a more precise measurement of the elliptical outline. Second, we drew the inter‐teardrop line as the horizontal reference and a line from the mid‐point of sacrococcygeal junction to the center of upper pole of symphysis pubis to define the pelvic radiographic axis (Figure 1). Then the Elliversion software automatically calculates the radiographic acetabulum cup inclination and two standardized Liaw's versions, defined as the angle between the pelvis radiographic axis and the plane of acetabulum with θ assigned to be positive (Liaw's version 1) and negative (Liaw's version 2), respectively.

### Determination of Anteversion or Retroversion

When measuring only one anteroposterior radiograph of the pelvis, it is impossible to determine which of the two values is correct. However, measuring two radiographs with different orientations and pelvis radiographic axes, despite small differences, allows us to identify the correct value. The correct value will only have a small difference between the two measurements due to measurement errors. Conversely, the incorrect value will have a larger difference between the two measurements. In other words, if the difference between the two measurements where θ is assigned as positive (indicating anteversion) is smaller than that assigned as negative (indicating retroversion), then anteversion is confirmed, and *vice versa* for retroversion.

## Results

**FIGURE 2:**
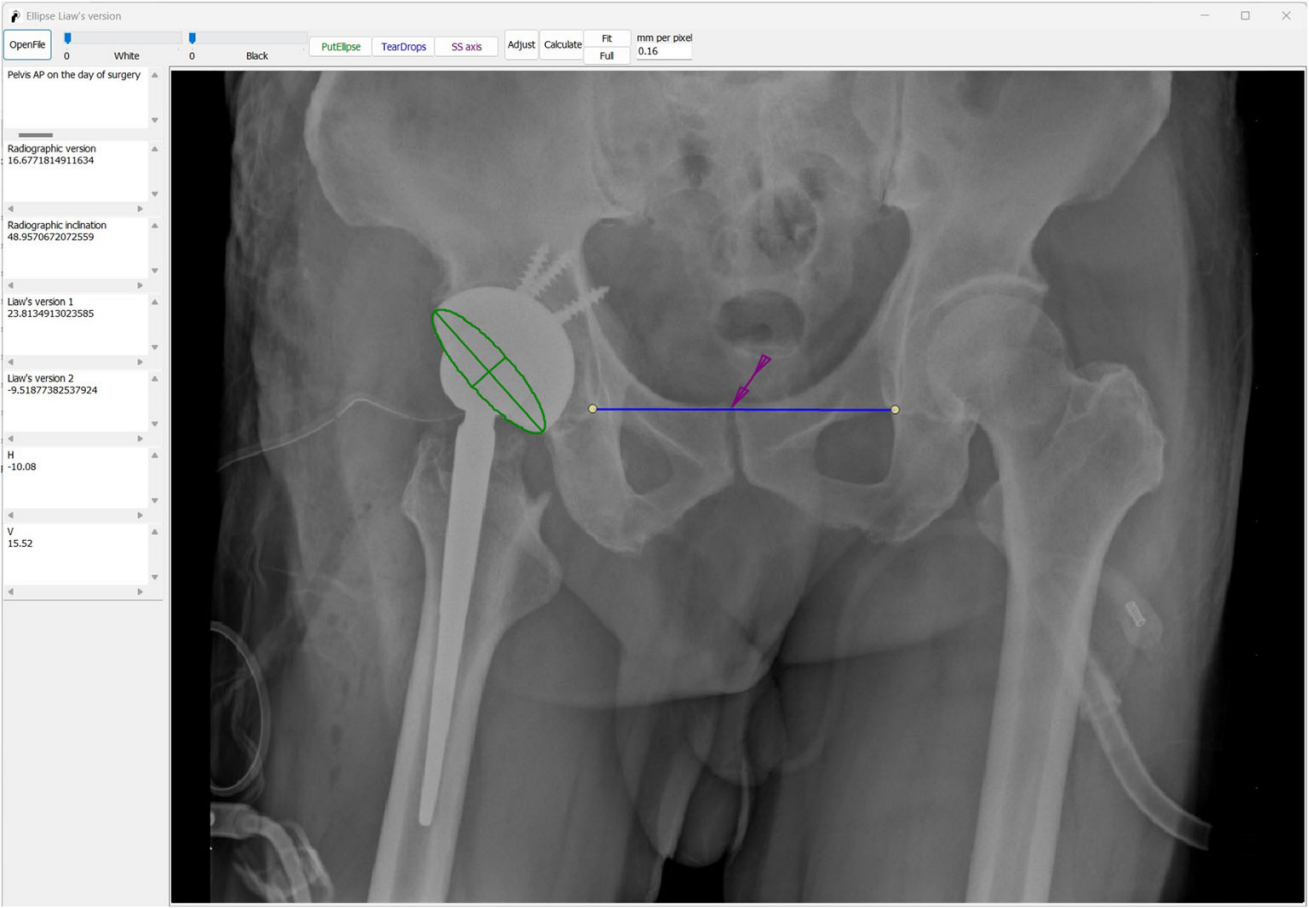
Pelvis anteroposterior radiographs on the operation day, the standardized anteversion was 23.81° (Liaw's version 1) when θ was assigned as positive (anteversion) and was −9.52° (Liaw's version 2) when θ was assigned as negative (retroversion).

**FIGURE 3:**
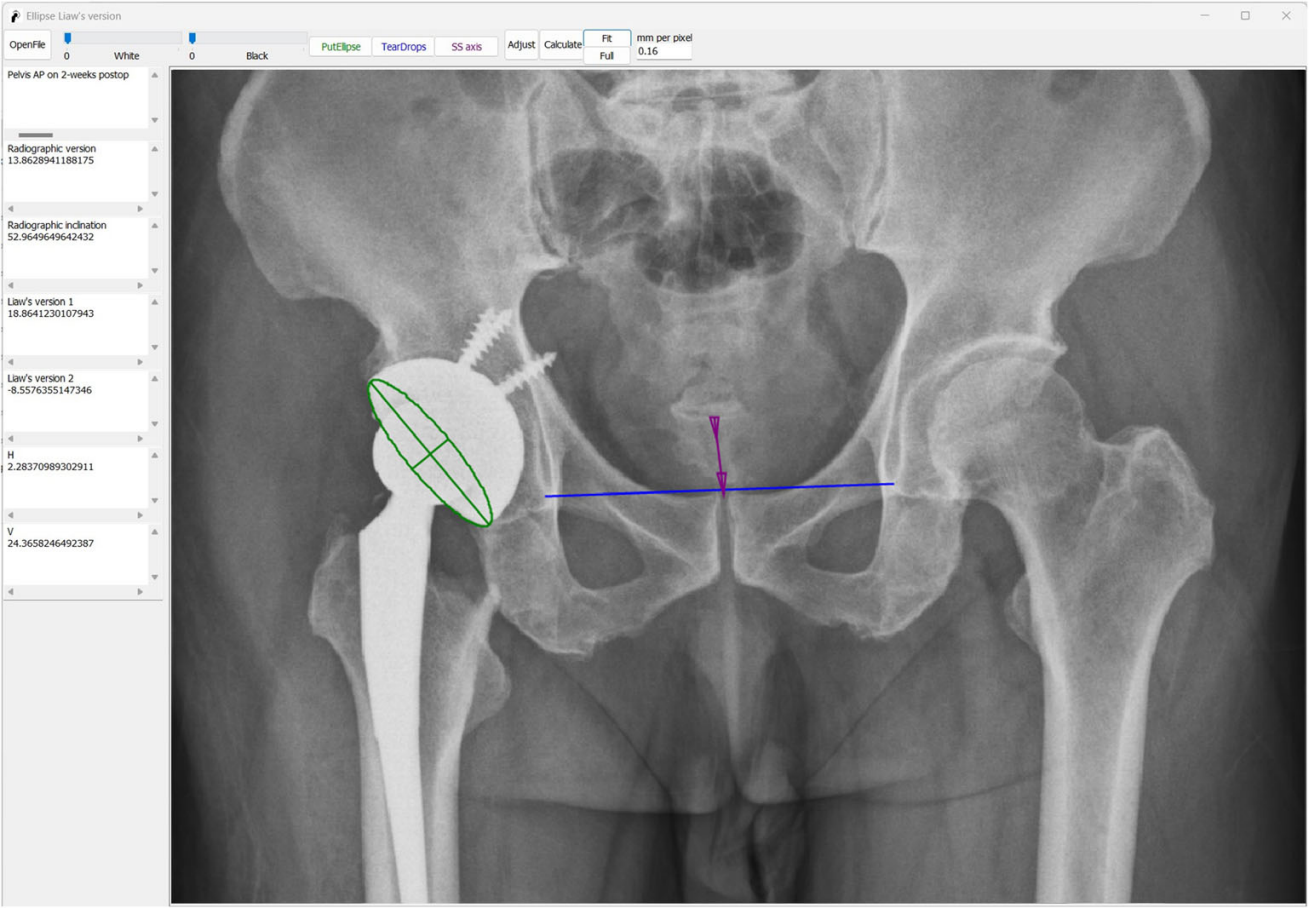
Pelvis anteroposterior radiographs at 2‐week follow up, the standardized anteversion was 18.86° (Liaw's version 1) when θ was assigned as positive (anteversion) and was −8.56° (Liaw's version 2) when θ was assigned as negative (retroversion).

**FIGURE 4:**
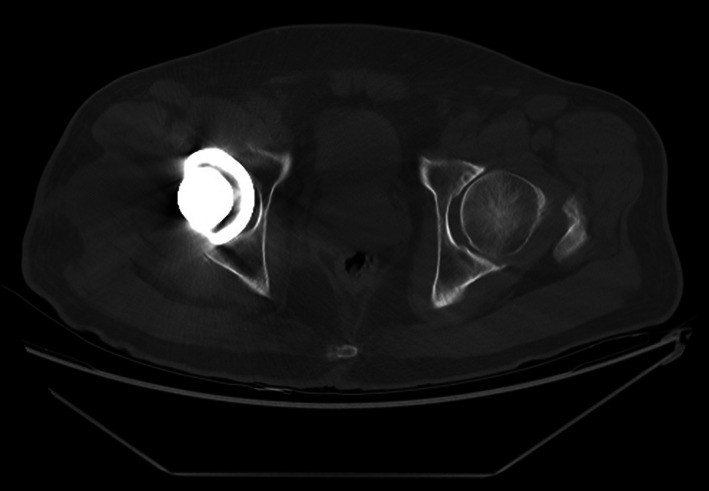
Axial view of pelvis CT performed at 6‐week follow‐up verified the retroverted acetabulum component.

The first author reviewed the postoperative pelvis anteroposterior radiographs taken on the day of the operation (Figure 2) and the 2‐week follow‐up (Figure 3). Acetabulum cup orientations were measured using the computerized ellipse method (Liaw's version) with the Elliversion software. Figure 2 showed that when θ was assigned as positive (anteversion), the standardized anteversion was measured as 23.81° (Liaw's version 1), and when θ was assigned as negative (retroversion), the standardized anteversion was measured as −9.52° (Liaw's version 2). In Figure 3, the standardized anteversion was 18.86° (Liaw's version 1) when θ was assigned as positive (anteversion) and was −8.56° (Liaw's version 2) when θ was assigned as negative (retroversion). We presumed that the acetabulum component was retroverted based on the comparison of measurements. The difference between the values of θ assigned as negative (9.52–8.56 = 0.96) was much smaller than the difference between the values of θ assigned as positive (23.81–18.86 = 4.95). The axial view of the computed tomography (CT) scan obtained at the 6‐week follow‐up verified the retroverted acetabulum component (Figure 4) with the anterior pelvic plane (APP) as the coronal plane reference.

## Discussion

While using the computerized ellipse method to measure Liaw's version improved accuracy and precision in measuring acetabular anteversion,^19^, ^20^ its ability to differentiate between anteversion and retroversion has yet to be confirmed. In this study, we demonstrate the application of computerized ellipse method for detecting and measuring acetabulum component retroversion. The version type is judged by the smaller difference in version angle assigned to be positive (anteversion) or negative (retroversion) between two measurements.

### Comparison with Other Two‐Dimensional Methods

Malpositioning of the acetabulum component is associated with postoperative dislocation,^3^, ^4^, ^5^, ^6^, ^7^ component loosening, accelerated polyethylene wear, poor range of motion, and impingement.^8^, ^9^, ^10^, ^11^ Thus, measuring the orientation of acetabulum component is a crucial postoperative follow‐up assessment for patients undergoing THA to predict function, prognosis, and susceptibility to dislocation. Several radiographic two‐dimensional methods have been described for measuring acetabulum component anteversion on plain radiographs.^3^, ^13^, ^14^, ^15^, ^16^, ^17^ However, none of these methods can differentiate between anteversion and retroversion. Traditionally, anteversion and retroversion can be distinguished by using either a cross‐table lateral view or by comparing the measured anteversion angle of two radiographs centered at the pubic symphysis (pelvic anteroposterior radiograph) and the hip (hip anteroposterior radiograph), respectively. However, these methods do not take pelvic flexion into account and may lead to misinterpretations. In contrast, the original standardized equation of Liaw's version took both pelvic flexion and anteversion or retroversion into consideration, achieved through the definition of the pelvis radiographic axis (from the mid‐point of the sacrococcygeal junction to the center of the upper pole of the symphysis pubis) and the standardization process.

### Comparison with CT‐Based Methods

Although CT‐based methods are currently widely used and easily accessible in clinical practice for determining acetabular component orientation after THA due to their relatively good intra‐ and interobserver reliability and the high level of accuracy,^21^, ^22^, ^23^ they have some drawbacks. The availability of scanning time, high radiation dose, and medical cost may deter routine or serial screening. Additionally, CT scanning may not be practical if it is not readily accessible and may not be suitable for retrospective reviews. Furthermore, cup orientation can still differ between standing and supine positions due to different pelvis flexion. In contrast, our proposed method offers several benefits, including low radiation exposure, low cost, no requirement for additional equipment, and a simple and highly accurate measurement process with the aid of the Elliversion software. This method can also be used for retrospective reviews as long as anteroposterior pelvic radiographs are available.

### Limitations

There are certain limitations to this study. Firstly, only one case was used for demonstration, which raises concerns about the validity of the method. However, this is due to the relative rarity of retroverted cases in clinical practice. The purpose of this brief report is to demonstrate how to utilize the computerized ellipse method to distinguish between anteversion and retroversion. Additionally, Liaw's version has some inherent limitations. Firstly, the standardization equation is based on the assumption that the pelvis is perfectly symmetrical. Therefore, some errors may occur when measuring the asymmetrical pelvis. Secondly, to discriminate between anteversion and retroversion of the acetabulum component, two high‐quality pelvic anteroposterior radiographs with different orientations and clear delineation of the pubic symphysis, sacrococcygeal junction, and bilateral teardrops are still necessary.

### Strengths and Prospect of Clinical Application

Regardless, to the best of our knowledge, this is the first two‐dimensional radiographic method that can distinguish retroversion from anteversion. It can be applied as a valuable tool for detecting acetabular component retroversion on anteroposterior radiographs during routine postoperative follow‐up and retrospective assessments of patients undergoing THA. However, a larger‐scale study with an increased number of cases should be conducted to provide further clarification regarding the reliability and accuracy of this method.

### Conclusion

Measuring the orientation of the acetabular component following THA is crucial for prognosis but can also be a cumbersome task for orthopaedic doctors. The computerized ellipse method not only provides a reliable measurement of the acetabulum anteversion angle, but also represents the first two‐dimensional radiographic method capable of distinguishing retroversion from anteversion by comparing measurements from two anteroposterior radiographs of the pelvis. The Elliversion software makes the measurement process quick and easy. We propose using the computerized ellipse method as a tool to detect acetabular component retroversion on anteroposterior radiographs during routine postoperative follow‐up and retrospective reviews of patients undergoing THA.

## Author Contributions

All authors had full access to the data in the study and take responsibility for the integrity of the data and the accuracy of the data analysis. *Conceptualization*, Chun‐Hao Lin and Chen‐Kun Liaw; *Methodology*, Chen‐Kun Liaw; Software, Chen‐Kun Liaw; Validation, Wei‐Cheng Chen; *Investigation*, Chun‐Hao Lin and Wei‐Cheng Chen; *Formal Analysis*, Chun‐Hao Lin and Wei‐Cheng Chen; *Resources*, Chen‐Kun Liaw; *Writing – Original Draft*, Chun‐Hao Lin; *Writing – Review & Editing*, Chun‐Hao Lin, Wei‐Cheng Chen, Pei‐Wei Weng, Yu‐min Huang and Chen‐Kun Liaw.; *Visualization*, Chun‐Hao Lin, Pei‐Wei Weng, Yu‐min Huang; *Supervision*, Chen‐Kun Liaw; *Funding Acquisition*, Chen‐Kun Liaw.

## Ethical Statement

This statement indicates that the study described in the document was approved by the Institutional Review Board of Taipei Medical University, Taiwan, with the reference number N202005040 and the approval date of May 15, 2020. It also indicates that patient consent to participate was waived because the study only used radiographic images of patients and did not involve any of their medical history information. This means that the researchers were granted permission to conduct the study without obtaining explicit consent from the patients, as the information gathered was considered non‐sensitive and did not pose any risk to their privacy or well‐being.

## FUNDING INFORMATION

This work was funded by grants from the Taiwan National Science and Technology Council (MOST109‐2314‐B‐038‐029), Bridge funding of Taipei Medical University (TMU108‐AE1‐B43), and other fundings of Taipei Medical University (TMU109‐Y05‐E173), and (TMU111‐F‐009).

## Conflict of Interest statement

The authors declare that all authors listed meet the authorship criteria according to the latest guidelines of the International Committee of Medical Journal Editors, and all authors reviewed the manuscript and approved its final version.

## Data Availability

The datasets used and analyzed during the current study are available from the corresponding author on reasonable requests. The Elliversion software can also be obtained from the corresponding author upon requests.
